# Colorectal cancer‐initiating cells caught in the act

**DOI:** 10.15252/emmm.201707858

**Published:** 2017-05-30

**Authors:** Sebastian M Dieter, Hanno Glimm, Claudia R Ball

**Affiliations:** ^1^ Department of Translational OncologyNational Center for Tumor Diseases (NCT) and German Cancer Research Center (DKFZ)HeidelbergGermany; ^2^ German Consortium for Translational Cancer Research (DKTK)HeidelbergGermany

**Keywords:** Cancer, Digestive System, Stem Cells

## Abstract

Our increased awareness of the clonal organization of many hematological and solid cancers has dramatically changed our view on the design of novel therapeutic approaches for cancer. Tumor‐initiating cells (TIC) (a.k.a. cancer stem cells) are on the apex in this hierarchy and can self‐renew and differentiate, thereby continuously fueling tumor growth and metastasis formation. This process was previously thought to be unidirectional. Self‐renewing TIC therefore represent highly attractive targets for therapeutic intervention.

Functional heterogeneity in CRC is somewhat akin to the organization of the normal intestinal stem cell (ISC) compartment, where crypt‐based columnar cells (CBCC) expressing the Wnt target gene LGR5 are responsible for tissue homeostasis and regeneration (Beumer & Clevers, 2016). CBCC reside at the crypt base interspersed with, and in close contact to postmitotic Paneth cells, which constitute the stem cell niche for these LGR5^+^ ISC (Sato *et al*, 2011). Compartmentalization of the intestinal crypt structure into a stem cell compartment at the base and a differentiated compartment at the intestinal villus or the colon surface is dependent on a Wnt gradient whose intensity is strongest at the crypt bottom. Active Wnt signaling is essential for stem cell maintenance and activity (Basu *et al*, 2016). It should therefore hold little surprise that the signaling pathway most affected in colon cancers is the Wnt pathway. Although the R‐spondin/LGR5/RNF43 signaling axis can also be directly mutated, the most frequent cause for deregulated Wnt signaling in CRC is loss of APC expression. Strikingly, in the absence of APC, LGR5^+^ cells can fuel tumor growth (Barker *et al*, 2009). Although accumulating evidence hints toward a major role of LGR5^+^ cells in the TIC compartment (Zhan *et al*, 2017), the lack of universal TIC markers, suitable LGR5 antibodies, or tracing systems has so far limited our ability to address the functional relevance of LGR5^+^ cells in human CRC. Three recent publications by Cortina *et al*, Shimokawa *et al*, and Melo *et al* (2017), from the Batlle, Sato, and de Sauvage groups, respectively*,* now strongly heighten our understanding of the essential role that LGR5^+^ cells play in CRC growth and metastasis based on genetic fate mapping.

**Figure 1:**
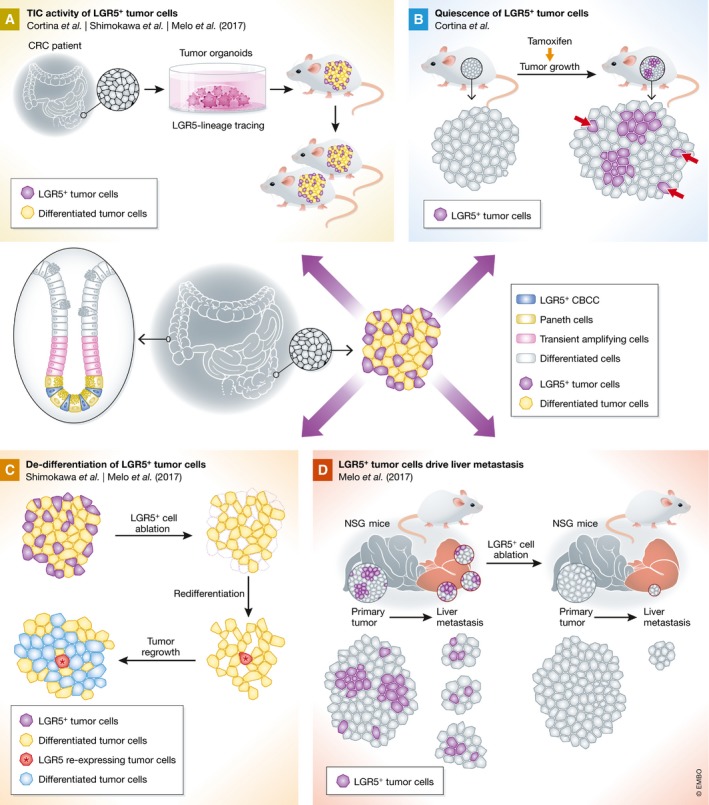
Utilizing lineage tracing, the Eduard Batlle (Cortina *et al*, 2017), Toshiro Sato (Shimokawa *et al*, 2017) and Frederic de Sauvage (Melo *et al*, 2017) groups demonstrate that LGR5^+^ cells are essential for tumor growth and metastasis formation of CRC (A) Lineage tracing of primary human CRC‐derived organoid cells or murine organoid cells mimicking human CRC demonstrates tumor‐initiating cell (TIC) activity of LGR5^+^ cells in serial transplantation. (B) Induction of LGR5‐lineage tracing upon initial tumor formation demonstrates the existence of quiescent LGR5^+^ tumor cells (red arrows). (C) Following ablation of LGR5^+^ cells (purple) in established tumors *in vivo*, differentiated tumor cells (yellow) can re‐express LGR5 (red) and drive tumor re‐growth (blue). (D) Upon Lgr5^+^ ablation in transplantation models of metastasis formation, liver metastasis burden decreased, whereas primary tumor growth is not affected.

As elegantly presented by Cortina *et al* in the current issue of *EMBO Molecular Medicine*, CRISPR/Cas9 can be used to transfer lineage‐tracing approaches to primary human cancer cells. Specifically, the authors used CRISPR/Cas9 to integrate EGFP reporter and lineage‐tracing cassettes into the LGR5 locus of human CRC organoids. LGR5‐EGFP^+^ cells could then be isolated from organoid‐derived xenografts and were shown to express gene programs similar to those of normal ISC. Moreover, LGR5‐EGFP^+^ cells were highly tumorigenic in recipient mice, indicating strong enrichment of TIC in the LGR5^+^ population. Lineage tracing of LGR5‐EGFP^+^ CRC cells in organoid‐derived xenografts demonstrated their self‐renewal and ability to differentiate into mucosecreting‐ and absorptive‐like phenotypes *in vivo*, thereby providing convincing evidence for LGR5 as a *bona fide* TIC marker in human CRC. Interestingly, a population of mitotically inactive LGR5^+^ cells remained quiescent for prolonged times. This is in line with the description of a dormant TIC population present in primary CRC xenografts identified by genetic barcoding (Dieter *et al*, 2011; Kreso *et al*, 2013), and suggesting that such a population could fuel tumor growth following ablation of actively dividing cells. Moreover, it will be interesting to understand whether resting LGR5^+^ cells are similar to the reserve stem cell populations in normal human intestinal epithelium, which are considered to intervene during regeneration (Beumer & Clevers, 2016). Given that organoid technology allows for manipulating normal and malignant ISC, the presented approach enables both directly comparing stem cell systems and assessing a potential therapeutic window based on selective gene dependencies.

The broad applicability of genetic fate mapping strategies is also exemplified in the two other recent publications. Shimokawa *et al* used CRISPR/Cas9 to insert a rainbow reporter into the LGR5 locus and then traced LGR5^+^ human CRC organoid cells in xenografts. In line with Cortina *et al,* they demonstrate TIC activity of LGR5^+^ cells. Next, they generated LGR5‐iCaspase9‐tdTomato organoids, allowing LGR5^+^ cell ablation upon dimerizer treatment. After successful LGR5 depletion, tumors shrank significantly but eventually regrew in parallel with LGR5 re‐expression, suggesting plasticity of LGR5^−^ tumor cells. Directing a lineage‐tracing cassette to the KRT20 locus to track differentiated cells demonstrated that KRT20^+^ cells can reverse into LGR5^+^ cells with TIC capacity upon LGR5^+^‐cell ablation, thereby driving tumor re‐growth.

This surprising finding was independently confirmed by Melo *et al* (2017) using engineered mouse models of intestinal tumorigenesis. Mice express the diphtheria toxin (DT) receptor fused to GFP under the control of the endogenous Lgr5 regulatory region to visualize Lgr5^+^ cells. Moreover, Lgr5^+^ cells can be selectively ablated by DT administration. The authors generated intestinal organoid models that recapitulate human CRC progression by sequentially introducing *Trp53* and *Smad4* mutations. In accord with Cortina *et al* and Shimokawa *et al*, they found enriched TIC capacity in Lgr5‐GFP^+^ tumor cell fractions. Notably, Lgr5^+^ tumor cell depletion did not cause tumor regression, but rather tumor stasis, while treatment discontinuation was followed by rapid tumor re‐growth. Even upon complete loss of all Lgr5‐GFP^+^ cells, tumors were able to regenerate Lgr5‐GFP^+^ cells to initial levels within 1 week. Interestingly, these findings were fully recapitulated in organoids *in vitro*, suggesting that Lgr5^+^ cell rescue is independent from tumor stroma. These results confirm the plasticity of LGR5^−^ tumor cells described by Shimokawa *et al*. Mechanistically, Melo *et al* (2017) suggest a role of interferon‐α response and activation of Myc signaling for induction of Lgr5^+^ cell recovery, based on transcriptome analysis of Lgr5^+^ cell‐depleted tumors.

As demonstrated earlier by genetic barcoding, CRC TIC are critical for metastasis formation (Dieter *et al*, 2011). Melo *et al* (2017) probed the requirement of Lgr5^+^ TIC for metastasis formation and maintenance. Depletion of Lgr5^+^ TIC significantly decreased established tumor burden at metastatic sites or their formation in different metastasis models, demonstrating that Lgr5^+^ tumor cells play an important role for metastasis initiation and maintenance. Surprisingly, Lgr5^+^ ablation did not affect primary tumor growth in the colonic mucosa, whereas liver metastases substantially shrank, suggesting that the microenvironment may be relevant for the function or recruitment of Lgr5^+^ cells at liver metastatic sites.

In summary, these exciting reports profoundly impact on our understanding of TIC hierarchies. Previously regarded as fixed populations, the functionally heterogeneous composition of a tumor bulk rather appears as a dynamic structure, where the loss of TIC can be compensated by reversal of differentiated cancer cells as required.

This raises several new fundamental questions. First, it remains to be clarified whether the reversal of differentiated cancer cells is a “democratic” process, whereby every cancer cell can become a TIC, or whether distinct cell subfractions can be recruited as has been shown for the normal intestinal epithelium. Moreover, the mechanisms contributing to the reversal process and inducing cancer stemness need to be deciphered in order to design therapeutic strategies that not only target TIC, but additionally interfere with their compensation mechanisms. Whether TIC eradication alone is sufficient to treat metastatic disease will have to be determined by analyzing metastatic sites beyond the liver and by understanding the role of different microenvironments in TIC compensation.

Collectively, these reports have revealed fascinating novel aspects of TIC biology, raised new questions to be answered and, importantly, presented the tools to address them. From a therapeutic perspective, it becomes evident that to succeed, future therapeutic strategies will need to target the mechanisms underlying TIC activity rather than TIC *per se*.
